# Implementation of the 7-1-7 target for detection, notification, and response to public health threats in five countries: a retrospective, observational study

**DOI:** 10.1016/S2214-109X(23)00133-X

**Published:** 2023-04-13

**Authors:** Aaron F Bochner, Issa Makumbi, Olaolu Aderinola, Aschalew Abayneh, Ralph Jetoh, Rahel L Yemanaberhan, Jenom S Danjuma, Francis T Lazaro, Hani J Mahmoud, Trokon O Yeabah, Lydia Nakiire, Aperki K Yahaya, Renato A Teixeira, Mohammed Lamorde, Immaculate Nabukenya, John Oladejo, Ifedayo M O Adetifa, Wanderson Oliveira, Amanda McClelland, Christopher T Lee

**Affiliations:** a Resolve to Save Lives, New York, NY, USA; b Republic of Uganda Ministry of Health, Kampala, Uganda; c Nigeria Centre for Disease Control and Prevention, Abuja, Nigeria; d Ethiopian Public Health Institute, Addis Ababa, Ethiopia; e National Public Health Institute of Liberia, Monrovia, Liberia; f Resolve to Save Lives Initiative, Vital Strategies, Addis Ababa, Ethiopia; g Infectious Diseases Institute, Makerere University, Kampala, Uganda; h Vital Strategies, São Paulo, Brazil; i Ministry of Defense Hospital of the Armed Forces, Brasília, Brazil

## Abstract

**Background:**

Suboptimal detection and response to recent outbreaks, including COVID-19 and mpox (formerly known as monkeypox), have shown that the world is insufficiently prepared for public health threats. Routine monitoring of detection and response performance of health emergency systems through timeliness metrics has been proposed to evaluate and improve outbreak preparedness and contain health threats early. We implemented 7-1-7 to measure the timeliness of detection (target of ≤7 days from emergence), notification (target of ≤1 day from detection), and completion of seven early response actions (target of ≤7 days from notification), and we identified bottlenecks to and enablers of system performance.

**Methods:**

In this retrospective, observational study, we conducted reviews of public health events in Brazil, Ethiopia, Liberia, Nigeria, and Uganda with staff from ministries of health and national public health institutes. For selected public health events occurring from Jan 1, 2018, to Dec 31, 2022, we calculated timeliness intervals for detection, notification, and early response actions, and synthesised identified bottlenecks and enablers. We mapped bottlenecks and enablers to Joint External Evaluation (second edition) indicators.

**Findings:**

Of 41 public health events assessed, 22 (54%) met a target of 7 days to detect (median 6 days [range 0–157]), 29 (71%) met a target of 1 day to notify (0 days [0–24]), and 20 (49%) met a target of 7 days to complete all early response actions (8 days [0–72]). 11 (27%) events met the complete 7-1-7 target, with variation among event types. 25 (61%) of 41 bottlenecks to and 27 (51%) of 53 enablers of detection were at the health facility level, with delays to notification (14 [44%] of 32 bottlenecks) and response (22 [39%] of 56 bottlenecks) most often at an intermediate public health (ie, municipal, district, county, state, or province) level. Rapid resource mobilisation for responses (six [9%] of 65 enablers) from the national level enabled faster responses.

**Interpretation:**

The 7-1-7 target is feasible to measure and to achieve, and assessment with this framework can identify areas for performance improvement and help prioritise national planning. Increased investments must be made at the health facility and intermediate public health levels for improved systems to detect, notify, and rapidly respond to emerging public health threats.

**Funding:**

Bill & Melinda Gates Foundation.

## Introduction

The COVID-19 pandemic showed the importance of systems to detect, notify, and respond to disease outbreaks early and effectively at local levels to avert health, economic, and social effects at national and global levels. The pandemic highlighted limitations of existing measures of such capabilities,^1^, ^2^ including the Joint External Evaluation (JEE) and Global Health Security Index, which use static measures of capacity rather than assessing how systems function in real-world conditions. The Independent Panel for Pandemic Preparedness and Response recommended “a fundamental reassessment which better aligns preparedness measurement with operational capacities in real-world stress situations, including the points at which coordination structures and decision-making may fail”.^3^ Similarly, the 2005 International Health Regulations (IHR) Review Committee's report highlighted the importance of early alert, notification, and response and recommended that WHO strengthen its tools and processes for assessing core capacities, including the use of functional assessments.^4^ These recommendations underscore the importance of assessing the real-world performance of the complex systems required for detection and response.

Health emergencies are complex events, and their timely detection and response require capabilities at multiple levels: patients must have access to and seek care when ill, diseases must be recognised and then confirmed, and results must be made available at all levels of the health system to initiate a response. Results from process mapping have shown that a systems approach—reviewing the step-by-step performance of systems under real-world conditions—identified bottlenecks that, once addressed, accelerated disease control during previous outbreaks.^5^

**Research in context**

**Evidence before this study**

We searched PubMed for articles published between database inception and June 1, 2022, which had, in the title or abstract, the terms “timeliness” and either “outbreak” or “epidemic”, and identified 409 unique articles. We supplemented the scholarly literature review with a grey literature review for material published between Jan 1, 2000, and June 1, 2022, including normative and technical guidance from WHO. Only papers published in English were included. Some groups have proposed using timeliness metrics to monitor performance of disease surveillance systems. However, these metrics have not previously been connected to simple and clear targets for advocacy and communication or incorporated into a performance improvement framework. Although several groups have published timeliness results, an absence of standardised timeliness metric definitions prevents comparisons or pooling of data across studies. One study evaluated timeliness of 296 outbreaks in the WHO African region and found that timeliness of detection improved from 2017 to 2019. A study in Nigeria used timeliness metrics to show that an intervention—establishment of a revolving outbreak investigation fund—led to improvements in response timeliness. However, these studies did not present consolidated bottlenecks or enablers or describe a performance improvement framework.

**Added value of this study**

We used specific targets for timeliness of detection, notification, and early response actions indicating response initiation to allow clear communication and to identify bottlenecks and enablers, which were then aggregated and analysed to identify common themes. Through implementation in five countries, we found that the 7-1-7 target provides a systems framework through which countries were able to assess their epidemic preparedness capabilities at community, health facility, intermediate, and national levels. We also found that use of the 7-1-7 target complements existing preparedness measures by identifying bottlenecks and enablers of response that are not captured by the Joint External Evaluation tool, providing supplemental information that is useful for prioritising activities in national action plans for health security.

**Implications of all the available evidence**

Given the ability of 7-1-7 implementation to quantify the real-world performance of detection, notification, and response systems and identify bottlenecks and enablers for timely action, other ministries of health and national public health institutes might be interested in implementing 7-1-7 to evaluate and improve their epidemic preparedness capabilities.

Increasing efforts have been made to use measures of real-world performance both to assess system functioning and to identify important gaps for performance improvement. Intra-action reviews and after-action reviews have been leveraged to identify performance gaps and inform remediation plans.^6^, ^7^, ^8^ WHO's Thirteenth General Programme of Work (GPW 13) is measured using the Triple Billion impact framework, with a key tracer indicator of timely detection, notification, and response to calculate system performance in health emergencies.^9^

7-1-7 has been proposed as a target for outbreak detection, notification, and early response, whereby every suspected outbreak is detected within 7 days of emergence and reported to public health authorities within 1 day of detection, and seven early response actions are completed within 7 days from reporting to public health authorities, indicating timely initiation of response.^10^

The 7-1-7 target and its application integrate several successful aspects from previous work, including the use of timeliness metrics,^11^ real-world events for learning and bottleneck analysis (from intra-action reviews and after-action reviews), application of a systems approach (from process mapping), and quantification of systems timeliness (from the GPW 13). 7-1-7 draws on existing timeliness metrics and simplifies their presentation for communication and advocacy, provides a framework for performance improvement, and supplements them with targets. Clear targets (eg, 90-90-90 for HIV) can create accountability frameworks and facilitate communication of challenges to key stakeholders who might lack visibility on the complex systems of health emergencies.^12^

We assessed the feasibility and utility of the 7-1-7 approach to evaluate systems performance for detection, notification, and response in five countries. We summarised retrospective event data from these countries to evaluate historical performance and identify bottlenecks to inform performance improvement.

## Methods

### Study setting

For this retrospective, observational study, we partnered with national public health institutes in Ethiopia, Liberia, and Nigeria; the Ministry of Health in Uganda; and the Ministry of Health as well as municipal and state health departments in Brazil to conduct retrospective reviews of events that had occurred between Jan 1, 2018, and Dec 31, 2022. In alignment with WHO's methods for measurement of timely detection, notification, and response,^13^ countries selected events meeting IHR criteria for serious public health events, as described in IHR Annex 2,^14^ which include notifiable events under the IHR and events not formally notifiable to WHO but for which the number of cases, deaths, or both is large for the given place, time, or population; the event has the potential for high public health impact; or external assistance is needed to detect, investigate, respond to, and control the current event or prevent new cases. Results were intended to be used to prioritise national planning. Thus, rather than selecting a random sample of events, countries used a purposive sampling approach to prioritise events for selection that represented their risk landscape with data available.

### Data collection

Countries conducted desk reviews of selected events to complete structured templates and supplemented existing outbreak, situation, and intra-action and after-action reviews with inputs from subnational and national-level responders. Government officials responsible for the detection and response to these events identified milestone dates (date of emergence [*t*_0_], date of detection [*t*_d_], date of notification [*t*_n_], and dates of each of seven early response actions [*t*_r1 – 7_]), as well as bottlenecks (conditions that delayed actions) and enablers (conditions that facilitated prompt actions). Definitions of timeliness milestones are provided in the panel and appendix (p 2).

Panel **7-1-7 timeliness milestones and definitions**

**Date of emergence**

• For endemic diseases, date on which a predetermined increase in case incidence over baseline rates occurred
• For non-endemic diseases, date on which the index case or first epidemiologically linked case first experienced symptoms
• For other public health events, date the threat first met criteria as a reportable event based on country reporting standards

**Date of detection**

• Date the event is first recorded by any source or in any system

**Date of notification**

• Date the event is first reported to a public health authority responsible for action

**Date of completion of early response actions**

• Date on which all applicable early response actions were completed:
  • Initiate investigation or deploy investigation or response team
  • Conduct epidemiological analysis of burden, severity, and risk factors, and perform initial risk assessment
  • Obtain laboratory confirmation of the outbreak aetiology
  • Initiate appropriate case management and infection prevention and control measures in health facilities
  • Initiate appropriate public health countermeasures in affected communities
  • Initiate appropriate risk communication and community engagement activities
  • Establish a coordination mechanism

During 2021–22, government officials responsible for detection and response to health events in each country convened for a 1-day workshop to validate findings from the desk reviews and build consensus on priority bottleneck areas that would benefit from performance improvement. Because data were collected from existing response documentation and did not include personally identifiable information, the Resolve to Save Lives ethical review committee determined that this activity did not constitute human subject research. In addition, the ministry of health, national public health institutes, health department, or appropriate ethical bodies or institutional review boards in each country reviewed the protocols, made a non-human subject research determination, and gave permission to publish aggregate data.

### Data analysis

We consolidated data for all selected events with complete information for dates of emergence, detection, and notification into Microsoft Excel. We used Stata (version 17.0) to synthesise median timeliness measures and calculate the proportion of events meeting targets for timely detection (*t*_d_ – *t*_0_ ≤7 days), notification (*t*_n_ – *t*_d_ ≤1 day), and early response. To identify bottlenecks and enablers, we used two measures and targets for effective early response: at least one of the seven response actions was initiated within 1 day of notification (min[*t*_r1 – 7_] – *t*_n_ ≤1 day); and all applicable early response actions indicating initiation were completed within 7 days of notification (max[*t*_r1 – 7_] – *t*_n_ ≤7 days). If data were missing for any of the seven early response actions, we used the latest available date in the timeline as the date of completion of early response actions. The full 7-1-7 target was met when an event did not exceed any of the three targets. The rationale for selection of these targets has been described previously.^10^

We categorised events into six groups (foodborne or waterborne pathogen diseases, vaccine-preventable diseases, vector-borne diseases, viral haemorrhagic fevers, respiratory diseases, and other events).^15^ Other events included disease transmission among animals and chemical poisoning in humans.

### Analysis of bottlenecks and enablers

We generated descriptive codes using a grounded theory approach^16^ based on 129 bottlenecks and 162 enablers identified (free text) by workshop participants. A primary round of coding generated 34 codes for bottlenecks and 32 codes for enablers, which were further collapsed into a final code bank^17^ containing 26 unique categories of bottlenecks and 30 categories of enablers. Additionally, we coded the level of the public health system at which the bottleneck or enabler was observed: the community or health facility level; intermediate (municipal, district, county, state, or province) level; or the national level. To understand the alignment between operational capabilities identified by participants and the relevant technical capacities in the JEE (second edition) tool,^18^ we mapped each of the free text bottlenecks and enablers to JEE indicators and the JEE score level (from 1 indicating lowest capacity to 5 indicating highest capacity) required to reach the level of capacity described in the bottleneck or enabler. Two raters (AFB, CTL, HJM, FTL, RAT, JSD, or RLY) scored each bottleneck and enabler, with discordant results discussed and scored via group consensus. We used the JEE tool because it contains more indicators (n=49) than the State Party Self-Assessment Annual Reporting (SPAR; 2018 edition tool; n=24).^19^ If insufficient detail was available to identify a corresponding JEE indicator, we coded the value as missing; if the bottleneck or enabler did not map to an existing indicator, it was coded as not applicable. If a bottleneck or enabler aligned with a capacity measured by a JEE indicator, but none of the qualitative attributes used to assign a score reflected the specific content of the bottleneck or enabler, we coded the score as not applicable. If attributes were relevant for two scoring levels, we coded the lower value for consistency.

### Role of the funding source

The funder of the study had no role in study design; collection, analysis, and interpretation of data; writing of the report; or the decision to submit for publication.

## Results

Of the 41 events assessed, 11 (27%) were viral haemorrhagic fever outbreaks (Rift Valley fever, Lassa fever, or Crimean–Congo haemorrhagic fever), ten (24%) were vaccine-preventable disease outbreaks (measles, polio, and yellow fever), six (15%) were respiratory disease outbreaks (COVID-19 or influenza A), five (12%) were vector-borne disease outbreaks (Rickettsial infection, Chagas disease, dengue, or chikungunya), five (12%) were foodborne or waterborne disease outbreaks (botulism, cholera, or other foodborne disease outbreak), and four (10%) were other events (disease in animals or chemical poisoning in humans; table 1).

**Table 1:** Median timeliness of detection, notification, and early response and proportion of events meeting 7-1-7 targets in Brazil, Ethiopia, Liberia, Nigeria, and Uganda, 2018–22

	**Public health events (n=41)**	**Detection (target: 7 days)**		**Notification (target: 1 day)**		**Completion of early response (target: 7 days)**		**7-1-7 (all targets [met target])**
		Median (range)	Met target	Median (range)	Met target	Median (range)	Met target	
Viral haemorrhagic fever	11 (27%)	6 (1–14)	7/11 (64%)	0 (0–2)	9/11 (82%)	3 (2–10)	9/11 (82%)	6/11 (55%)
Vaccine-preventable	10 (24%)	16 (1–157)	3/10 (30%)	0 (0–15)	6/10 (60%)	31 (4–72)	1/10 (10%)	0
Respiratory	6 (15%)	4 (0–14)	4/6 (67%)	0 (0–1)	6/6 (100%)	4 (1–11)	4/6 (67%)	3/6 (50%)
Foodborne or waterborne	5 (12%)	1 (1–20)	4/5 (80%)	0 (0–4)	3/5 (60%)	17 (9–28)	0	0
Vector-borne	5 (12%)	38 (3–133)	1/5 (20%)	0 (0–17)	3/5 (60%)	4 (0–13)	4/5 (80%)	1/5 (20%)
Other events*	4 (10%)	2 (0–67)	3/4 (75%)	2 (0–24)	2/4 (50%)	7 (4–13)	2/4 (50%)	1/4 (25%)
All	41 (100%)	6 (0–157)	22/41 (54%)	0 (0–24)	29/41 (71%)	8 (0–72)	20/41 (49%)	11/41 (27%)

Data are n (%) or n/N (%) unless otherwise stated.

* Includes outbreak in animals and chemical poisoning in humans.

The median time to detection was 6 days (range 0–157; IQR 3–19). Detection was slowest for vector-borne diseases (median 38 days [range 3–133; IQR 20–45]) and vaccine-preventable diseases (median 16 days [range 1–157; IQR 6–31]; table 1). The median time to notify public health authorities was 0 days (range 0–24; IQR 0–2) across all event types, except for events outside the human health sector (median 2 days [range 0–24; IQR 0–8]; table 1). All seven early response actions were completed within a median of 8 days (range 0–72; IQR 4–15) and were slowest for vaccine-preventable diseases (median 31 days [range 4–72; IQR 17–44]) and foodborne or waterborne disease outbreaks (median 17 days [range 9–28; IQR 15–18]; table 1). 22 (54%) events met the target for detection, 29 (71%) met the target for notification, and 20 (49%) met the target for completion of early response actions. 11 (27%) events met all three components of the 7-1-7 target (table 1). The first response action was completed in a median of 0 days (IQR 0–1), with 33 (80%) events initiating responses within 1 day of notification.

We identified 41 bottlenecks to detection, 32 bottlenecks to notification, and 56 bottlenecks to response, and 53 enablers of detection, 44 enablers of notification, and 65 enablers of response (table 2). 25 (61%) of 41 bottlenecks to and 27 (51%) of 53 enablers of detection were at the health facility or community level. The most frequently observed bottleneck to detection was low awareness or clinical suspicion by health workers (12 [29%]), followed by delays in laboratory confirmation (four [10%]; table 3). The most frequent enabler of detection was strong clinical suspicion or health worker awareness of the case definition (11 [21%]), followed by having clinical surveillance focal points with the capacity to report to the public health system (six [11%]; table 4).

**Table 2:** Levels of the public health system at which bottlenecks and enablers are observed

		**Bottlenecks**	**Enablers**
Detection		41	53
	Health facility or community	25/41 (61%)	27/53 (51%)
	Intermediate*	9/41 (22%)	21/53 (40%)
	National	3/41 (7%)	4/53 (8%)
	Multiple levels	3/41 (7%)	1/53 (2%)
	Unknown	1/41 (2%)	0
Notification		32	44
	Health facility or community	9/32 (28%)	11/44 (25%)
	Intermediate*	14/32 (44%)	17/44 (39%)
	National	7/32 (22%)	12/44 (27%)
	Multiple levels	1/32 (3%)	4/44 (9%)
	Unknown	1/32 (3%)	0
Response		56	65
	Health facility or community	12/56 (21%)	6/65 (9%)
	Intermediate*	22/56 (39%)	25/65 (38%)
	National	19/56 (34%)	29/65 (45%)
	Multiple levels	2/56 (4%)	4/65 (6%)
	Unknown	1/56 (2%)	1/65 (2%)

Data are n or n/N (%).

* Municipal, district, county, state, or province.

**Table 3:** Bottlenecks to detection, notification, and response

	**Detection (n=41)**	**Notification (n=32)**	**Response (n=56)**	**Total (n=129)**
Laboratory confirmation	4 (10%)	2 (6%)	7 (13%)	13 (10%)
Low awareness or clinical suspicion by health workers	12 (29%)	0	0	12 (9%)
Availability of resources for response initiation or rapid resource mobilisation	0	1 (3%)	9 (16%)	10 (8%)
Reporting failure	1 (2%)	7 (22%)	1 (2%)	9 (7%)
Access issues (event occurred in remote, fragile, or conflict settings)	2 (5%)	1 (3%)	5 (9%)	8 (6%)
Competing priorities (including COVID-19)	3 (7%)	0	5 (9%)	8 (6%)
Human resources gaps for public health	1 (2%)	3 (9%)	3 (5%)	7 (5%)
Limited availability of countermeasures or personal protective equipment	0	0	7 (13%)	7 (5%)
Multi-agency coordination	0	2 (6%)	4 (7%)	6 (5%)
Technological challenge for electronic surveillance or reporting systems (eg, network coverage)	1 (2%)	4 (13%)	0	5 (4%)
Clinical surveillance focal point or capacity	1 (2%)	2 (6%)	1 (2%)	4 (3%)
Delayed specimen collection	3 (7%)	1 (3%)	0	4 (3%)
Low community knowledge or trust	2 (5%)	0	2 (4%)	4 (3%)
One health information sharing or collaboration (eg, between human health and animal health)	2 (5%)	1 (3%)	1 (2%)	4 (3%)
Specimen transportation	0	1 (3%)	3 (5%)	4 (3%)
Weak response coordination, including incident management and rapid response team capacity	0	1 (3%)	3 (5%)	4 (3%)
Data entry delay	2 (5%)	1 (3%)	0	3 (2%)
Failure to conduct early risk assessment or event verification	1 (2%)	2 (6%)	0	3 (2%)
New or unexpected pathogen	1 (2%)	2 (6%)	0	3 (2%)
Sensitivity of community detection	3 (7%)	0	0	3 (2%)
Delay in care seeking by patients	2 (5%)	0	0	2 (2%)
Insufficient clinical case management capacity	0	0	2 (4%)	2 (2%)
Inadequate risk assessments or preparedness plans	0	0	1 (2%)	1 (1%)
Scarcity of diagnostic commodities (laboratory reagents, rapid diagnostic tests, or specimen collection kits)	0	1 (3%)	0	1 (1%)
Logistics and shipment delays	0	0	1 (2%)	1 (1%)
Risk communications or community engagement	0	0	1 (2%)	1 (1%)

Data are n (%).

**Table 4:** Enablers of detection, notification, and response

	**Detection (n=53)**	**Notification (n=44)**	**Response (n=65)**	**Total (n=162)**
Clinical surveillance focal point or capacity	6 (11%)	6 (14%)	2 (3%)	14 (9%)
Adequate and trained public health workforce	1 (2%)	5 (11%)	5 (8%)	11 (7%)
Strong clinical suspicion or awareness of case definition by health workers	11 (21%)	0	0	11 (7%)
Strong reporting lines for public health surveillance	2 (4%)	8 (18%)	1 (2%)	11 (7%)
Rapid coordinated response mechanism in place	1 (2%)	1 (2%)	8 (12%)	10 (6%)
Specimen transportation	5 (9%)	0	4 (6%)	9 (6%)
Multi-sector and multi-stakeholder collaboration (including partners)	1 (2%)	1 (2%)	6 (9%)	8 (5%)
Feedback systems for laboratory results in place	1 (2%)	6 (14%)	0	7 (4%)
Availability of resources for response initiation or rapid resource mobilisation	0	0	6 (9%)	6 (4%)
Emergency operations centre or incident management capacity for preparedness and response	1 (2%)	0	5 (8%)	6 (4%)
Laboratory diagnostic capability	0	3 (7%)	3 (5%)	6 (4%)
Availability of medical countermeasures	0	0	5 (8%)	5 (3%)
Prompt specimen collection	3 (6%)	0	2 (3%)	5 (3%)
Multi-sector or multidisciplinary response team mechanisms in place	0	0	5 (8%)	5 (3%)
Community engagement and trust of public health system	2 (4%)	1 (2%)	1 (2%)	4 (2%)
Coordination and communication between clinical and public health systems	3 (6%)	1 (2%)	0	4 (2%)
Mobile internet network coverage	0	4 (9%)	0	4 (2%)
Operational readiness plans implemented	1 (2%)	1 (2%)	2 (3%)	4 (2%)
Synthesis, integration, and use of data for action	2 (4%)	2 (5%)	0	4 (2%)
Availability of diagnostic commodities (laboratory reagents, rapid diagnostic tests, or specimen collection kits)	2 (4%)	0	1 (2%)	3 (2%)
Availability of event-based surveillance system	1 (2%)	2 (5%)	0	3 (2%)
Case management capacity	1 (2%)	0	2 (3%)	3 (2%)
Cross-border or points of entry public health capacity	1 (2%)	0	2 (3%)	3 (2%)
Existing animal health surveillance system	2 (4%)	1 (2%)	0	3 (2%)
Functional indicator-based surveillance in place	2 (4%)	0	1 (2%)	3 (2%)
Rapid response team deployment mechanism in place	1 (2%)	0	2 (3%)	3 (2%)
Active or sentinel surveillance	2 (4%)	0	0	2 (1%)
Electronic surveillance or reporting system	0	2 (5%)	0	2 (1%)
Resources in animal health sector	0	0	2 (3%)	2 (1%)
Environmental surveillance system	1 (2%)	0	0	1 (1%)

Data are n (%).

14 (44%) of 32 bottlenecks and 17 (39%) of 44 enablers for notification were at the intermediate level (table 2). The most common bottlenecks to notification were reporting failure (ie, the party detecting an event did not communicate to the relevant authority; seven [22%]) and technological challenges for electronic surveillance or reporting systems (four [13%]; table 3). The most common enablers of notification were strong reporting lines for public health (eight [18%]), having clinical surveillance focal points with the capacity to report to the public health system (six [14%]), and the existence of feedback systems for laboratory results (six [14%]; table 4).

Although response bottlenecks were most frequently observed at the intermediate level (22 [39%] of 56), enablers were most frequently observed at the national level (29 [45%] of 65; table 2). The most common bottlenecks to response were unavailability of resources for rapid response initiation or resource mobilisation (nine [16%]), delayed laboratory confirmation (seven [13%]), and limited availability or access to countermeasures including medication, vaccine, and personal protective equipment (seven [13%]; table 3). The most common enablers for response were rapid coordinated response mechanisms in place (eight [12%]), availability of resources for rapid response initiation or resource mobilisation (six [9%]), and multi-sector and multi-stakeholder collaboration (six [9%]; table 4).

Of 291 total bottlenecks and enablers observed, 248 (85%) could be crosswalked to existing JEE indicators, particularly those for reporting channels (45), case management procedures including health facility implementation of case definitions and standard operating procedures (31), availability of human resources (22), laboratory diagnostic capacity (17), and funding availability for timely response to health emergencies (16; table 5). Three (1%) bottlenecks or enablers did not have sufficient information to assign a JEE indicator, and 40 (12%) bottlenecks or enablers were not represented by JEE indicators, including access issues (conflict or remote settings; eight), COVID-19-related prioritisation challenges (eight), technological challenges including lack of mobile network coverage (seven), and low community knowledge or trust in the public health system (four).

**Table 5:** Crosswalk of identified bottlenecks and enablers with the JEE (second edition) indicators and score

	**Frequency**	**Median score**	Frequency of not applicable*score
P.1.3 A financing mechanism and funds are available for timely response to health emergencies	16	4	0
P.4.1 Coordinated surveillance systems in place in the animal health and public health sectors for zoonotic diseases and pathogens identified as joint priorities	6	4	1
P.4.2 Mechanisms for responding to infectious and potential zoonotic diseases established and functional	14	4	1
P.5.1 Surveillance systems in place for the detection and monitoring of foodborne diseases and food contamination	1	4	0
P.5.2 Mechanisms are established and functioning for the response and management of food safety emergencies	1	4	0
P.7.2 National vaccine access and delivery	6	3	0
D.1.1 Laboratory testing for detection of priority diseases	17	4	11
D.1.2 Specimen referral and transport system	13	3	1
D.1.3 Effective national diagnostic network	3	3	0
D.2.1 Surveillance systems	14	4	5
D.2.2 Use of electronic tools	5	4	2
D.2.3 Analysis of surveillance data	4	4	1
D.3.1 System for efficient reporting to FAO, WOAH (formerly OIE), and WHO	1	3	0
D.3.2 Reporting network and protocols in country	45	4	0
D.4.2 Human resources are available to effectively implement IHR	22	4	0
D.4.3 In-service trainings are available	1	4	0
D.4.4 FETP or other applied epidemiology training programme is in place	2	3	0
R.1.1 Strategy emergency risk assessments conducted and emergency resources identified and mapped	1	4	0
R.1.2 National multi-sectoral multihazard emergency preparedness measures, including emergency response plans, are developed, implemented, and tested	3	4	1
R.2.1 Emergency response coordination	12	4	1
R.2.2 Emergency operations centre capacities, procedures, and plans	3	4	0
R.3.1 Linking public health and security authorities	3	5	0
R.4.1 System in place for activating and coordinating medical countermeasures during a public health emergency	9	..	9
R.4.2 System in place for activating and coordinating health personnel during a public health emergency	7	..	7
R.4.3 Case management procedures implemented for IHR relevant hazards	31	4	0
R.5.3 Public communication for emergencies	1	3	0
R.5.4 Communication engagement with affected communities	1	4	0
R.5.5 Addressing perceptions, risky behaviours, and misinformation	1	4	0
POE.1 Routine capacities established at points of entry	3	2	0
POE.2 Effective public health response at points of entry	2	3	0
Not captured by existing JEE indicator	40	..	..
Insufficient detail to identify a corresponding JEE indicator	3	..	..
Total	291	4	40

FAO=Food and Agriculture Organization. FETP=Field Epidemiology Training Program. IHR=International Health Regulations. JEE=Joint External Evaluation. OIE=Office International des Epizooties. WOAH=World Organisation for Animal Health.

* Not applicable score indicates that although the bottleneck or enabler is captured by the indicator description, the score attributes in the JEE (second edition) tool would not measure the existence of the capability described by the bottleneck or enabler.

Of the 248 bottlenecks and enablers that aligned with a JEE indicator, 40 (16%) could not be assigned a score because JEE scoring criteria did not capture the specified capability. Of the 208 bottlenecks and enablers that could be assigned a JEE score, 169 (81%) required a relatively high score (4 or 5) for sufficient capacity to achieve timely detection, notification, and response.

## Discussion

In this retrospective review of public health events in five countries, we applied the 7-1-7 target to measure country capabilities for detection, notification, and early response initiation. We found that the median performance across all events suggests strong performance (6 days for detection, 0 days for notification, and 8 days to complete early response actions), but only a minority of events met all targets. Bottlenecks were most common at the subnational level, including the health facility level. Use of the 7-1-7 approach can supplement existing preparedness measures by identifying operational gaps from real-world events that might not be represented in the JEE (eg, community knowledge or trust in public health systems, and access issues) and can support prioritisation of national planning.

Timeliness metrics have previously been used to identify gaps and demonstrate improvements in timeliness of disease detection and reporting.^11^, ^15^, ^20^ However, fewer studies report on timeliness of response to events, and there is no indication of progress on outbreak response and mitigation. Countries have used timeliness data to review disease surveillance system performance. A multi-country effort in the Mekong basin analysed data from 2087 outbreaks and found that dates of index onset, report, and response were more than 95% complete in all countries.^21^ Similarly, a study from Nigeria used timeliness data (time to detection, notification, and response activities) to identify bottlenecks in detection and response to a *Neisseria meningitidis* serogroup C outbreak.^22^

In this study, we describe the use of a simplified metric for reporting on timeliness that aligns with and supports implementation of the WHO Triple Billion impact framework. The 7-1-7 target was designed to align with and support implementation of the IHR, specifically capacities described in Annex 1 at the community or primary public health response level, intermediate public health response level, and national level.^14^ The early response actions supplement the IHR Annex 1 to distinguish early response efforts from capacities required for extended responses for larger events.^10^ Although we found that early responses were initiated within 1 day for most events (80%), fewer than half of events met the 7-day target for completion of all seven actions that comprise effective response initiation. Prompt response initiation was necessary but not sufficient for an effective early response. The 7-1-7 target highlights the importance of initiating a response coordinated across multiple pillars and can be used to assess the effectiveness of surveillance and response systems (eg, integrated disease surveillance and response in the WHO African region), as well as identify and advocate for implementation of appropriate performance improvement measures.

For this analysis, countries conducted retrospective reviews of events that represented their risk landscapes. The approach to bottleneck and enabler identification and classification can be replicated in intra-action reviews, after-action reviews, and simulation exercises using the timeliness metrics described in WHO's country implementation guidance to review performance during the early phase of an outbreak or emergency and prioritise recommendations.^23^ These metrics and bottlenecks can inform development and prioritisation of national planning, including national action plans for health security and multi-sector development plans. Because 7-1-7 evaluates the performance of systems involved in health threat detection and response and generates recommendations that should be translated into national planning and advocacy, its implementation is best domiciled in a relevant public health agency with the mandate for surveillance, response, and preparedness planning (eg, IHR national focal point).

Prospective implementation of 7-1-7 can reduce the retrospective data collection burden and guide real-time performance management for ongoing events.^5^ Data collection should ideally be integrated into event management systems,^24^ but can also be collected in response coordination tools or situation reports. Identification of bottlenecks and enablers can be best documented by the teams involved in the initial event investigation and response, either within rapid response team reports or through review of performance of an ongoing or a recent event against the 7-1-7 target. Teams can mitigate desirability bias by adhering to 7-1-7 milestone definitions and using a self-assessment approach (similar to SPAR and after-action reviews) focusing on identifying bottlenecks to achieve performance improvement.

Our analysis indicates that the 7-1-7 target is achievable across pathogen types and highlights the need for continued system strengthening. Most event types met at least two of the three targets, apart from vaccine-preventable diseases. Although substantial improvements have been made in surveillance and response for viral haemorrhagic fever outbreaks, functional capacities for surveillance need to be strengthened particularly for vaccine-preventable and vector-borne diseases, for which early responses can guide the targeted deployment of countermeasures and accelerate goals towards elimination or eradication. In addition, the finding that detection targets were not met for 33% of respiratory events (COVID-19 or influenza A) highlights that there are gaps in preparedness even for anticipated events and shows the value of the 7-1-7 target for both anticipated and unanticipated events. The event types for which notification and response delays were longest often required coordination of multiple sectors (eg, animal-borne or vector-borne disease events) or multiple units within the ministry of health (eg, vaccine-preventable or foodborne or waterborne diseases). The findings highlight the importance of operationalising multi-sectoral coordination mechanisms for public health events that include routine communication channels for sharing epidemic intelligence and incident management protocols for multi-sectoral response governance.

Five pilot countries have used 7-1-7 to inform performance improvement efforts at national and subnational levels. Assessing actual performance during an event identified operational gaps that might not have been identified or prioritised for improvement by existing tools and metrics and integrated priority actions into national operational plans. Their experiences show that investments in disease detection must increase at the health facility level, where most events are detected by clinicians outside the public health system. Clear communication and reporting channels between health workers and surveillance officers are crucial to verify events and initiate a larger public health response.

We found that response bottlenecks most frequently involved resource limitations, including flexible funds for deployment of teams and availability of countermeasures, at the subnational level. National level resource availability to augment these gaps was a frequent enabler. In Nigeria, establishment of a flexible and rapid funding mechanism for early outbreak investigation decreased the median time to respond from 6 days to 2 days.^25^ Implementation of 7-1-7 by countries at national and intermediate public health levels might similarly identify catalytic investments to detect and respond to public health events more quickly.

Our approach has limitations. First, 7-1-7 measures a subset of systems required for preparedness—those required for early detection and early response rather than for later-stage or larger-scale responses—and does not include the development, production, or ability to scale implementation of countermeasures or to prevent the initial emergence of dangerous pathogens. Second, our retrospective review methodology was restricted by historic data availability, limiting the number of assessed events, and self-assessment might have introduced desirability bias. The non-random sample of events prohibits further statistical inference, although we found that descriptive analyses identified common themes that may suggest potential root causes. Last, the countries involved in piloting the 7-1-7 approach are not a representative global sample and we are unable to generalise their performance or their observed bottlenecks.

As next steps, we propose and have initiated research in three areas for future evaluation. First, several more countries have initiated prospective implementation of 7-1-7 to capture data on all serious public health events, which will create a more representative dataset and generate a larger sample of events that would allow for disaggregated analyses of correlates of 7-1-7 performance by pathogen type and transmission scenario, improving our understanding of representativeness and generalisability. Second, we will evaluate 7-1-7 performance characteristics by assessing internal validity and inter-rater reliability of timeliness measures. Last, we propose an evaluation of associations between timeliness and impact metrics, such as morbidity and mortality, to assess external validity of the target.

We have found value in implementing 7-1-7 as both a performance metric and a systems tool for performance improvement. We recommend its adoption for real-time monitoring and performance evaluation during public health events, with routine synthesis of data to track progress on the functioning of systems required for early detection and action and to initiate and target rapid quality improvement processes. Adopting 7-1-7 as a monitoring and evaluation tool at national level can help countries to prioritise investments and capacity-building actions and to support measurement of global progress through the GPW 13 and regional integrated surveillance strategies. We have found that 7-1-7 implementation can also engage stakeholders outside of the health emergencies sector and identify clear targets for joint decision making and advocacy for more rapid detection and response to health threats.

## Data sharing

Data requests can be channelled to each participating country by the corresponding author (CTL).

## Declaration of interests

We declare no competing interests.
