# Supplementary material for: Implementation of the 7-1-7 target for detection, notification, and response to public health threats in five countries: a retrospective, observational study
Source: Lancet Glob Health. 2023 Apr 13;11(6):e871–9. doi: 10.1016/S2214-109X(23)00133-X (PMC10156425; doi:10.1016/S2214-109X(23)00133-X)
Supplement: Supplementary appendix [file mmc1.pdf]

THE LANCET
Global Health

Supplementary appendix

This appendix formed part of the original submission and has been peer reviewed. We post it as supplied by the authors.

Supplement to: Bochner AF, Makumbi I, Aderinola O, et al. Implementation of the 7-1-7 target for detection, notification, and response to public health threats in five countries: a retrospective, observational study. *Lancet Glob Health* 2023; published online April 12. https://doi.org/10.1016/S2214-109X(23)00133-X.

7-1-7 ASSESSMENT TOOL

Rapid performance improvement for outbreak detection and response

Background

The emergence of SARS-CoV-2 and the resultant COVID-19 pandemic have demonstrated the importance of timely and effective systems to detect, notify and respond to outbreaks before they become epidemics. The pandemic has highlighted limitations in existing measures of health security capacity and underscores the importance of evaluating not only the presence, but also the performance of the complex systems required for early detection and response.

Timeliness metrics for outbreak detection, notification and response can be used to assess system performance. With clear targets for each milestone, these metrics can inform real-time performance gaps by surfacing bottlenecks where targets are not being met. Building on the International Health Regulations (2005) and World Health Organization's "triple billion" methodology, 7-1-7 metrics simplify performance evaluation, provide a blueprint for outbreak communication and drive performance improvement: every suspected outbreak should be detected within 7 days of emergence, with public health authorities notified within 1 day of detection, and initiated all early response actions within 7 days, including the following:

- Initiate investigation or deploy investigation/response team
- Conduct epidemiologic analysis of burden, severity and risk factors, and perform initial risk assessment
- Obtain laboratory confirmation of the outbreak etiology
- Initiate appropriate case management and infection prevention and control (IPC) measures in health facilities
- Initiate appropriate public health countermeasures in affected communities
- Initiate appropriate risk communication and community engagement activities
- Establish a coordination mechanism

Objective

Assess systems performance for detection, notification and response for an outbreak or other public health event, and identify bottlenecks and enablers for rapid performance improvement.

Methods

Proposed methods outlined in this tool can be applied at any scale — from local administrative units to national health authorities. Follow Steps 1-4 to document and report 7-1-7 metrics and recommendations.

Documentation

Step 1. Record dates for milestones

**Milestones**	**Date** DD/MM/YY	**Narrative** Briefly describe key observations in this interval and how the date was determined.
**Date of emergence**[1]<br>*For endemic diseases:*<br>date on which a predetermined increase in case incidence over baseline rates occurred<br>*For non-endemic diseases:*<br>date on which the index case or first epidemiologically linked case first experienced symptoms<br>*For other public health events:*<br>date the threat first met criteria as a reportable event based on country reporting standards		
**Date of detection**<br>Date the event is first recorded by any source or in any system		
**Date of notification**<br>Date the event is first reported to a public health authority responsible for action		
**Date of early response initiation**<br>Date on which the first of the seven early response actions occurred (see below)		
**Date of early response completion**<br>Date on which all applicable early response actions were completed (see below)		

**Early response actions**<br>Reference this list to determine the dates of early response initiation and completion.	**Date**<br>DD/MM/YY or N/A
Initiate investigation or deploy investigation/response team	
Conduct epidemiologic analysis of burden, severity and risk factors, and perform initial risk assessment	
Obtain laboratory confirmation of the outbreak etiology	
Initiate appropriate case management and infection prevention and control (IPC) measures in health facilities	
Initiate appropriate public health countermeasures[2] in affected communities	
Initiate appropriate risk communication and community engagement activities	
Establish a coordination mechanism	

[1] Date of emergence may change as data are updated through the course of the epidemiologic investigation.
[2] Procurement and distribution of commodities in the community to prevent outbreak spread (e.g., vaccines, ORS sachets, antimicrobial agents, water treatment, soap, insect repellants, bed nets, PPE), initiation of public health and social measures (e.g., masking, travel restrictions, quarantine, food recall, boil water advisory)

Step 2. Calculate timeliness in 7-1-7 intervals

Interval	Calculation<br>In days	Timeliness<br>In days	Target<br>In days	Met target?<br>Yes/No
**Detection**	Difference between dates of emergence and detection		**7**	
**Notification**	Difference between dates of detection and notification		**1**	
**Response**	Difference between dates of notification and completion of the last early response action		**7**	

Step 3. Identify bottlenecks and enablers

Interval	**Bottlenecks**<br>Factors that prevented timely action. Identify max 3, if applicable. Propose remedial actions in Step 4.	**Enablers**<br>Factors that enabled timely action. Identify max 3, if applicable. Document for advocacy and to demonstrate impact.
**Detection**		
**Notification**		
**Response**		

Step 4. Propose remedial actions to address bottlenecks

Immediate actions

Actions for immediate implementation (e.g., where resources are available or anticipated).

Proposed action	Bottleneck addressed	Responsible authority	Target start date	Target end date

Longer-term actions

Actions for longer-term planning and funding (e.g., through planning and budget cycles)

Proposed action	Bottleneck addressed	Responsible authority	Opportunities for planning and funding (e.g., incorporate in NAPHS, funding proposals)
